# *Esteya Vermicola*, a Nematophagous Fungus Attacking the Pine Wood Nematode, Harbors a Bacterial Endosymbiont Affiliated with *Gammaproteobacteria*

**DOI:** 10.1264/jsme2.ME16167

**Published:** 2017-09-27

**Authors:** Ruizhen Wang, Leiming Dong, Yuequ Chen, Liangjian Qu, Qinghua Wang, Yongan Zhang

**Affiliations:** 1 The Key Laboratory of Forest Protection, State Forestry Administration of China, Research Institute of Forest Ecology, Environment and Protection, Chinese Academy of Forestry China; 2 State Key Laboratory of Tree Genetics and Breeding, Research Institute of Forestry, Chinese Academy of Forestry China; 3 Forestry Resources Protection Institute, Jilin Provincial Academy of Forestry Sciences China

**Keywords:** symbiosis, endobacteria, *Esteya vermicola*

## Abstract

Symbioses have played pivotal roles in biological, ecological, and evolutionary diversification. Symbiotic bacteria affect the biology of hosts in a number of ways. *Esteya vermicola*, an endoparasitic nematophagous fungus, has high infectivity in the pine wood nematode (PWN), which causes devastating ecological damage and economic losses in Asia and Europe. An integration of molecular, phylogenetic, and morphological analyses revealed that surface-sterilized *E. vermicola* with septate hyphae from different geographic locations harbor bacterial endosymbionts. 16S rRNA gene sequences from four fungal strains all clustered in a well-supported monophyletic clade that was the most closely related to *Pseudomonas stutzeri* and affiliated with *Gammaproteobacteria*. The existence and intracellular location of endobacteria was revealed by fluorescent *in situ* hybridization (FISH). Our results showed that endobacteria were coccoid, vertically inherited, as yet uncultured, and essential symbionts. Ultrastructural observations indicated that young and old endobacteria differed in cell size, cell wall thickness, and the degree of reproduction. The results of the present study provide a fundamental understanding of the endobacteria inside *E. vermicola* and raise questions regarding the impact of endobacteria on the biology, ecology, and evolution of their fungal host.

Symbioses have played pivotal roles in biological, ecological, and evolutionary diversification (2, 29), and are crucial to the lifestyles of animals, plants, fungi, and prokaryotes (18). Symbiotic interactions play many important functions in their hosts: symbionts supply nutrition for the host; affect reproduction, development, and behavior; have various defensive roles against parasitoids, parasites, predators, and pathogens, some involving toxins or up-regulation of the host’s immunity (27, 29, 37); and some result in expanded ecological capabilities (33). These symbionts are very similar to organelles in the extent of their genetic and physiological integration with their hosts and also their extreme genomic reduction (33).

Bacterial endosymbionts are widespread among animals in nature (29, 56) and symbioses between bacteria and insects have been investigated in depth (13). However, limited information is currently available on fungal endobacteria. The endobacteria of *Glomeromycota* have been investigated in detail since the early 1970s (34). The symbiosis of *Glomeromycota* with *Glomeribacter* is suggested to be at least 400 million years old (32). Two AMF endobacteria may be distinguished based on their morphological features: a coccoid bacterium phylogenetically related to *Mollicutes* displaying a homogeneous Gram-positive-like wall structure with a wide distribution across *Glomeromycota* (36); and a rod-shaped, Gram-negative beta-proteobacterium phylogenetically related to *Burkholderia* (8), ‘*Candidatus* Glomeribacter gigasporarum’ (*Ca*Gg), common in several species of the family *Gigasporaceae* (7, 32). Endobacteria play an important role in fungal biology and ecology. Although the endobacterium *Ca*Gg exhibits extreme dependence on its host for nutrients and energy, it improves AMF sporulation success and fungal bioenergetic capacity, which contribute to fungal ecological fitness (44). Partida-Martinez *et al.* proved that the virulence of the fungal host *Rhizopus microsporus* to rice seeding was increased by endosymbionts because toxic rhizonin and rhizoxin are produced by *Burkholderia* endosymbionts and not by the fungus host (38, 39). *Burkholderia* endosymbionts have also been shown to master the sporulation capabilities of its host (40).

Most fungi possessing symbiotic bacteria are linked to the phyla *Glomeromycota* (6, 7, 35, 36, 50) and *Zygomycota* including *Rhizopus* (21, 38, 40) and *Mortierella* (20, 45). Nevertheless, septate fungi that host endobacteria have been discovered, such as *Tuber borchii* of *Ascomycota* (3), and *Laccaria bicolori* (4) and *Sebacinales* of *Basidiomycota* (47).

*E. vermicola*, which was examined in the present study, is an endoparasitic nematophagous fungus that attacks the pine wood nematode (PWN, *Bursaphelenchus xylophilus*) (25). The PWN is responsible for an epidemic of pine wilt disease, which causes severe ecological and economic losses in Asia and Europe (14). Adhesive lunate conidia produced by *E. vermicola* attach to and penetrate the cuticle of the PWN and consume the contents of the infected nematode’s body, and new lunate conidia are then produced using the PWN cadaver for the next infection cycle (53–55). Ninety percent of inoculated PWN are infected by adhesive conidia within 24 h, and almost all nematodes are killed within 8–10 d of inoculation (25, 53). Its high infectivity in the PWN suggests that *E. vermicola* has the potential to be a biocontrol agent (25, 55); however, the mechanisms underlying infection currently remain unclear.

Limited information is currently available on the endosymbionts of biocontrol fungi, particularly nematophagous fungi. Initial observations of *E. vermicola* by TEM indicated that symbiotic bacteria existed in the cytoplasm of *E. vermicola*. Endosymbionts may be important to the biology and ecology of biological control fungi. In order to confirm the presence of endobacteria and identify and assess their distribution in diverse isolated strains of *E. vermicola*, we investigated endosymbionts in four isolates originating from four countries and two continents. We combined transmission electron microscopy (TEM), scanning electron microscopy (SEM), and confocal microscopy to clarify the ultrastructural morphology and localization of endobacteria in the *E. vermicola* cytoplasm, as well as sequencing and phylogenetic analyses to reveal the endosymbiont’s phylogeny. We herein demonstrated that the septate fungus *E. vermicola* hosts endobacteria. The discovery of the endobacteria as important components of nematophagous fungi provides new insights into the multiple complexity of interphylum interactions among *E. vermicola*, its endobacteria, and the PWN.

## Materials and Methods

### Biological materials

*E. vermicola* strains (CBS100821, ATCC74485, CBS115803, and CNU120806) were used for experiments. Six strains of *E. vermicola* have been identified worldwide. Please refer to research advances on *E. vermicola* for further details of isolation substrates and origins (9).

### DNA extraction, amplification, and clone library analysis

Four *E. vermicola* strains were cultured in potato dextrose broth (PDB) at 25°C for 7 d with shaking. Fungal hyphae and spores were filtered, frozen in liquid nitrogen, and ground to a fine powder. DNA was isolated from the fine powder using the Qiagen Blood and Cell Culture DNA Kit (catalog no. Mini 13323) (Qiagen, Hilden, Germany) according to the instruction manual in an aseptic environment. Bacterial 16S rRNA genes were amplified using the universal bacterial primer pair 27f (5′-GAGAGTTTGATCCTGGCTCAG-3′) and 1492r (5′-TACGGYTACCTTGTTACGACTT-3′). PCR amplification was performed in a Gene Amp PCR System 2400 (Perkin-Elmer, Massachusetts, USA) in a total volume of 50 μL containing 0.25 μL Phusion High-Fidelity DNA Polymerase EX-TaKaRa *Taq* (TaKaRa, Shiga, Japan) (5 U μL^−1^), 5 μL 10×PCR buffer (Mg^2+^ Plus), 4 μL dNTP mixture (2.5 mM), 1 μL of each primer (20 μM), and 2.5 ng DNA. An initial denaturation step at 94°C for 5 min was followed by 30 cycles of denaturation at 94°C for 30 s, primer annealing at 60°C for 45 s, and elongation at 72°C for 90 s; with a final extension at 72°C for 10 min. PCR products from bacterial amplification were cloned using the pGEM-t Easy Vector System (Promega, Madison, USA) and then transformed into One Shot Top10 Chemically Competent *Escherichia coli* (Invitrogen, California, USA). Ninety colonies were sequenced and screened for insert lengths by PCR. Bacterial clones were sequenced on an ABI 3730xl automated sequencer (Applied Biosystems, California, USA).

### Phylogenetic analysis

Phylogenetic trees were constructed by analyzing the data of 16S rRNA gene sequences of endobacteria inside fungi living in diversity of environment and of >99% identity from blast hits of target sequence from GenBank. The alignment of selected sequences was performed using the SILVA web aligner (http://www.arb-silva.de/aligner/) (42) with default settings, except that bases remaining unaligned at the ends were removed. Any column that contained only gap characters in the resulting alignment was filtered out using MOTHUR (46). Phylogenetic trees were inferred with maximum likelihood (ML) and Bayesian inference (BI). The ML analysis was performed with RAxML ver. 8.2.4 (“BlackBox” version) (49) using automatic bootstraps and the GTRGAMMAI model for both bootstrapping and tree inference through the CIPRES Science Gateway web portal (31). The BI analysis using the same GTRGAMMAI model was performed with MrBayes ver. 3.2.6 (43), also run on CIPRES. The inference consisted of 6,755,000 generations (stopped automatically due to the convergence of all parameters) with sampling every 5,000 generations. The first 25% of samples were discarded as burn-in. Tree results were viewed and edited using the program FigTree v1.4.2 (http://tree.bio.ed.ac.uk/software/figtree/).

### Isolation and cultivation of bacterial symbionts

Surface-sterilized hyphae were ground in a homogenizer and resuspended in 0.85% NaCl (w/v). The suspension was centrifuged at 1,000 rpm at 4°C for 2 min. The supernatant was filtered with a 5-μm filter (Sartorius, Göttingen, Germany), then centrifuged at 10,000 rpm at 4°C for 20 min. The supernatant was removed gently to avoid losing the pellet; the pellet was resuspended in 0.85% NaCl (w/v). The suspension was plated onto agar plates and incubated at 25°C. Cultivability tests were performed with the purified bacteria obtained from the above steps. The five media used in the tests were LB, trypticase soy agar (TSA; per L: 5 g soy peptone, 3 g yeast extract, 15 g agar), marine yeast medium (per L: 24.0 g NaCl, 20.0 g agar, 5.0 g yeast extract, 20.0 mL 1 M phosphate buffer [pH 6.8], 20.0 mL Hutner’s mineral base, and 7.0 mL 1N KOH; pH 6.8±0.2 at 25°C), SW-LB medium (per L: 10 g Bacto Tryptone, 5 g yeast extract, 15 g agar [all from Difco Laboratories, Detroit, MI, USA]), and nitrate medium (per L: 0.8 g K_2_HPO_4_·3H_2_O, 0.2 g KH_2_PO_4_, 0.1 g CaCl_2_, 0.5 g MgSO_4_·7H_2_O, 1.5 g [NH_4_]_2_SO_4_, 3.0 g yeast extract, 10.0 g KNO_3_, 10.0 g glycerin, and 15 g agar; pH adjusted to 7.2). Bacterial growth was checked after a 3-week incubation by looking for colonies on solid media. All operations were conducted in a sterile environment.

### Treatment of *E. vermicola* with antibiotics

In order to eliminate the intracellular bacterial endosymbiont and cure the fungus CBS115803, twenty-one antibiotics (Cefotaxime, Ceftazidime, Azlocillin, Ticarcillin, Oxacillin, Mezlocillin, Penicillin G, Gentamicin, Kanamycin, Neomycin, Tobramycin, Streptomycin, Amikacin, Ciprofloxacin, Levofloxacin, Nalidixic acid, Doxycycline, Minocycline, Tetracycline, Clindamycin, and Rifampicin), two processed objects (hyphae and spores), and two working concentrations (working concentration and 10×working concentration) were used. Plates were prepared with PDA supplemented with an antibiotic (Amresco, OH, USA). Young growing hyphae were transferred to freshly prepared plates containing an antibiotic every 4 d for a total of 6 times. Spores from PDA+antibiotic plates (7 d old) were transferred to a submerged culture of PDB with an antibiotic (working concentration and 10×working concentration) and incubated with 120 rpm orbital shaking at 25°C for 3 d. Spores were cultured with a continuous antibiotic treatment by replacing with new PDB+antibiotic every 3 d for a total of 6 times. This procedure was repeated three times, and the complete elimination of bacteria was assessed by microscopic examinations and a PCR analysis.

### Localization of endobacteria by fluorescence

#### Fluorescence *in situ* hybridization

One-week-old fungal cultures grown in PDA were fixed by adding 4% paraformaldehyde buffered with phosphate-buffered saline (PH 7.2) and an incubation at 4°C for 12 h. Thereafter, cultures were washed three times in 1×PBS. Fixed fungal material was dehydrated in an increasing ethanol series (50%, 80%, and 96% [v/v], 3 min each). Oligonucleotide probes were designed with ARB (5′-CACTCATCC GCTCGACTTGCATGTGTTAGG-3′). They matched the bacterial sequences amplified from *E. vermicola* and were fluorescently labeled with Cy3. The probe was synthesized in Sangon Biotech (Sangon Biotech, Shanghai, China). The probe was diluted with sterile water according to the manufacturer’s instructions. FISH was performed as described by Monica Sharma *et al.* (47). Before observations, slides were mounted in antifading reagent (Molecular Probes, Eugene, OR, USA), and were viewed using the confocal laser scanning microscopy LSCM Leica TCS SP5 (Leica, Wetzlar, Germany).

#### Fluorescent nucleic acid stains

Microscopic examinations were undertaken with fluorescent nucleic acid stains to clarify whether the small intercellular bodies observed within hyphae were bacteria. *E. vermicola* cultures were stained with the LIVE/DEAD BacLight Bacterial Viability Kit L7012 (Molecular Probes). According to the manufacturer’s instructions, 3 μL dye mixture was added per mL fungal suspension. The suspension and dye were mixed thoroughly and incubated at room temperature in the dark for 15 min. Stained mycelia were viewed using the confocal laser scanning microscopy LSCM Leica TCS SP8/SP2.

#### Limulus amebocyte lysate (LAL) test

The Limulus amebocyte lysate (LAL) test was performed using the mycelia of four *E. vermicola* strains (CBS100821, ATCC74485, CBS115803, and CNU120806) to detect bacterial endotoxin (Et). Mycelia grown (approximately 0.2 g) at 25°C for 7 d in PDB were collected and placed into Pyrogen-Free sterilized tubes containing 500 μL of sterilized MilliQ water. The sample was broken in TissueLyser II (Qiagen) for 25 min. One hundred microliters of supernatants were transferred to a new tube, and was diluted to three concentration gradients by 10 fold. Et was assessed using LAL test kits (Horseshoe Crab Reagent, Xiamen, China) according to the manufacturer’s instructions. Absorbance was measured at a wavelength of 545 nm on the BECKMAN counter DU800 (Beckman Coulter, CA, USA). Et concentrations were calculated based on the standard Et solution. All equipment used in this procedure was sterilized at 250°C for 30 min by dry heat sterilizers.

### Ultrastructural morphology of bacterial symbionts

#### TEM

Fungi (hyphae and spores) from PDA cultures (7 d old) were used for TEM observations. After 2.5% (v/v) glutaraldehyde fixation at 4°C for 6 h and 1% (w/v) OsO_4_ fixation at room temperature for 2 h (rinsing the specimen using pH 7.2 phosphate buffer five times for 7 min once after each fixation) and dehydration (50%, 70%, 85%, 95%, and 100% [v/v] acetone for 15 min, then 100% acetone [dried with CaCl_2_] twice at 4°C for 20 min), samples were infiltrated at 4°C with a 2:1 (v/v) mixture of acetone/LR White resin for 2 h, a 1:2 (v/v) mixture of acetone/LR White resin for 2 h, and twice with 100% LR White resin for 12 h. Ultrathin sections were stained with uranyl acetate and lead citrate. The samples prepared were observed on a JEOL JEM-1400 microscope (JEOL, Tokyo, Japan).

#### SEM

Isolated bacteria were fixed in 1.5 mL 2.5% (v/v) glutaraldehyde prepared in 0.1 M cacodylic acid buffer (pH 7.3) and incubated at 4°C overnight. After the fixation step, samples were rinsed in 0.1 M cacodylic acid buffer (pH 7.3) to remove the excess fixative, first at 4°C once for 10 min, then three times for 20 min. In order to obtain the fracture surface of endobacteria, the specimen above was quickly frozen in liquid nitrogen and was broken using TissueLyser II (Qiagen). The specimen was then dehydrated. The dehydration process included the immersion of specimens at 4°C in 50% (v/v) acetone for 5 min, 70% (v/v) acetone for 10 min, 80% (v/v) acetone for 10 min, 90% (v/v) acetone for 15 min, and 100% acetone (dried with CaCl_2_) twice for 20 min. They were then critical point dried with CO_2_ as a transitional fluid and finally sputter coated with gold-palladium using a JEOL JFC 1100 ion-sputtering device (JEOL). Bacteria were observed and photographed with a Hitachi S4800 microscope (Hitachi, Tokyo, Japan).

Sequence data were deposited at the NCBI database under the accession numbers KU761532, KU761533, KU761534, and KU761535.

## Results

### Identity of endobacteria

**Fig. 1:**
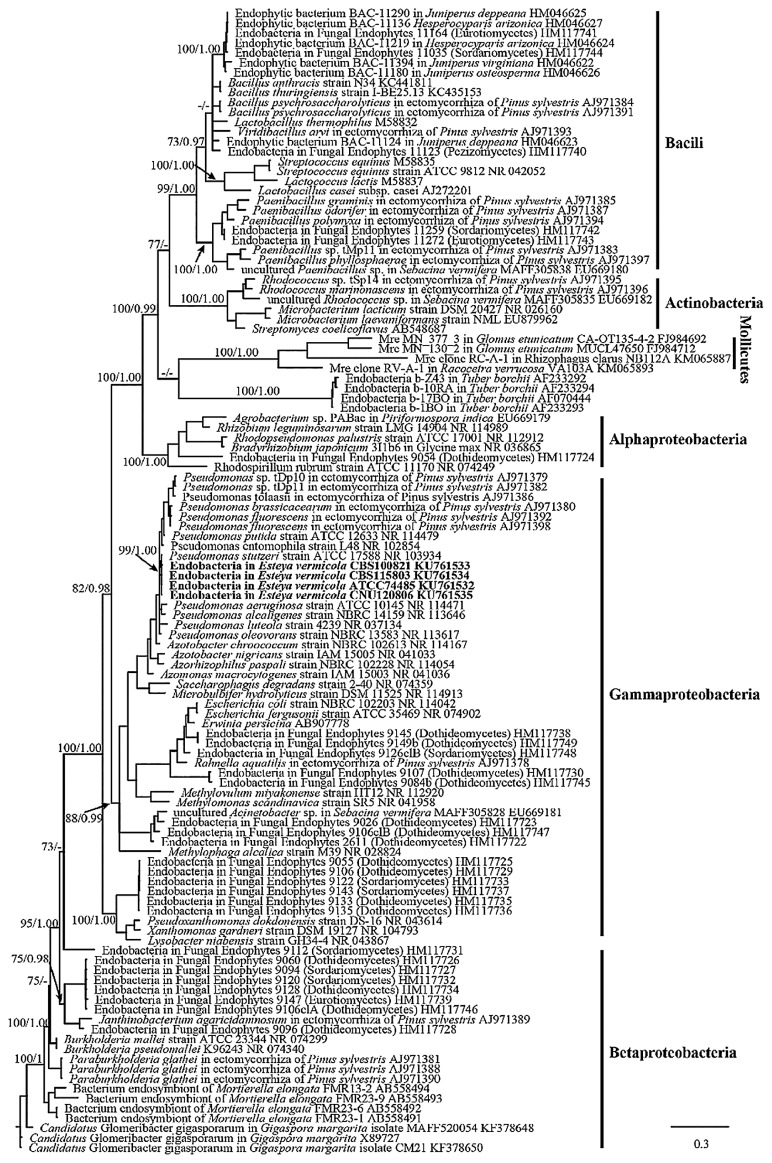
Phylogenetic placement of endobacterial 16S rRNA gene sequences. The DNA sequences retrieved in this work are shown in bold. 16S of endobacteria form diverse fungi were mainly selected as comparison sequence to infer their genetic relationship. Branches with ML bootstrap support (before the /) over 70% and Bayesian posterior probabilities (after the /) more than 0.95 were shown along branches.

Bacterial 16S rRNA gene sequences were amplified by PCR using a universal bacterial primer pair and sequenced to detect the presence of endosymbionts and analyze phylogenetic affiliations. PCR experiments revealed that endobacteria were always present in the surface-sterilized hypha and spores of four strains from different geographic locations (Fig. S1 and S2). The sequences reported in the present study (1,501 bp) have been deposited in the GenBank database. Within four given fungi species, only identical 16S rDNA sequences were obtained, indicating that the populations contained a single bacterial type. Database searches with the sequences obtained indicated the highest identity to *Pseudomonas* spp. (99% identical) sequences in the NCBI database. Furthermore, a phylogenetic analysis based on 16S rDNA revealed that endobacteria sequences from four fungal strains of different geographic regions all clustered in a well-supported monophyletic clade, most closely related to *P. stutzeri* and affiliated to *Gammaproteobacteria* (Fig. 1). Since endobacteria were broadly present inside four different *E. vermicola* strains, their occurrence was not an accidental or sporadic phenomenon.

**Fig. 2:**
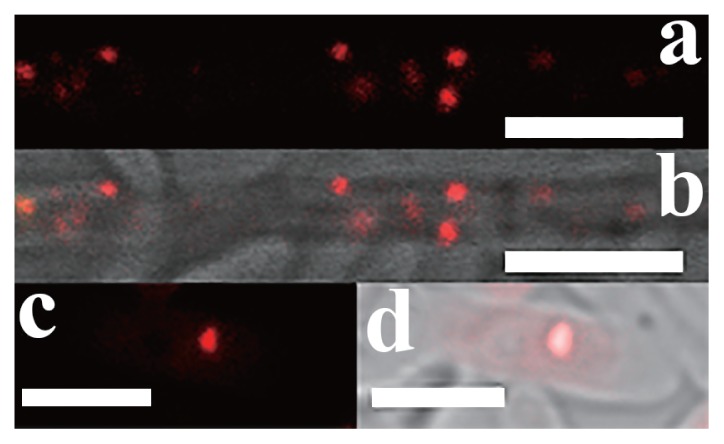
FISH on conidia and hyphae of *E. vermicola* CBS115803. (a, b) hypha, scale bar, 5 μm. (c, d) Lunate conidia, scale bar, 5 μm. a and c were dark field images; b and d were merged images of bright field images and dark field images. Endobacteria are seen as coccoid fluorescent spots in the cytoplasm of hypha and conidia, their 16S rRNA hybridized to the designed probe (red). Red fluorescent signals were endobacteria.

In order to confirm that bacteria were from inside fungi and identify their location, the fungal main reproductive organs, two kinds of conidia of *E. vermicola*, and hyphae were stained using fluorescence *in situ* hybridization. The 16S rDNA sequences in the present study were used to develop a probe to detect *Pseudomonas*. A fluorescence signal was not detected in any spore surface. Endobacteria presented coccoid fluorescent spots in the cytoplasm of conidia and hyphae. FISH indicated that the signals stained by Cy3 were endobacteria (Fig. 2).

In order to further prove the existence of endobacteria, Et experiments were also performed. The Et values of four *E. vermicola* strains were 242.09 EU g^−1^ wet mycelia (CBS115803), 209.12 EU g^−1^ wet mycelia (CNU120806), 215.69 EU g^−1^ wet mycelia (CBS100821), and 220.80 EU g^−1^ wet mycelia (ATCC 74485). These results showed that all four fungal strains were endobacterium-positive strains.

### Cultivation and elimination of endobacteria inside

E. vermicola The purified endobacteria isolated from *E. vermicola* were cultured in five different culture media at 25°C for 1 month under sterile conditions in order to investigate the free-living capacities of *E. vermicola* endobacteria. Five media were suitable for sustaining the growth of *Pseudomonas* spp.. However, endobacterial growth was never observed in any of the media tested.

With the aim of eliminating the endobacteria inside *E. vermicola* and curing the fungi, *E. vermicola* was cultivated on potato dextrose (PDA and PDB) in the presence of an antibiotic. Since the 16S rRNA analysis verified that bacterial symbionts were the most closely related to *P. stutzeri*, the selected antibiotics were effective against free-living *P. stutzeri* (23). However, after the axenic culturing of *E. vermicola* at 25°C for 1 month in the presence of antibiotics, bacteria were not eliminated from hyphae or spores, as confirmed by microscopic examinations and PCR analyses (Fig. not shown). Although tobramycin partly inhibited the growth of *E. vermicola* in the early stage, endobacteria continued to thrive in the host. To date, attempts to grow endobacteria and treatments to cure *E. vermicola* have been unsuccessful.

### Ultrastructural morphology of bacterial symbionts

**Fig. 3:**
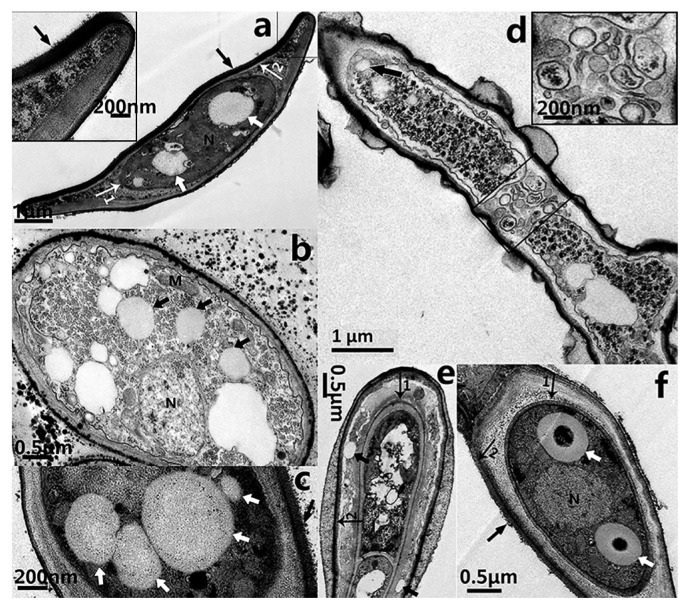
Transmission electron microscope images of *E. vermicola* CBS115803. (a) The endobacteria (white arrow) were present in the lunate spores (here cut in a longitudinal section). The inset showed part of the adhesion layer enlarged. (b) The endobacteria (black arrow) was thriving in hyphae. (c) The endobacteria (white arrow) inside lunate spore (with adhesive layer, black arrow). (d) Endobacteria arrived hyphal tip before septal formation. Rectangle showed the forming septum (black arrow, endobacteria). The inset showed the forming septum enlarged. (e, f) The forming lunate spore. (e) During the beginning of lunate sporulation, endobacteria (black arrow) present between inner cellwall (arrow 1) and outer cellwall (arrow 2). (f) The endobacteria (white arrrows) were enclosed inside the inner cellwall of maturing spores. The lunate spore that connects conidiogenous cells was maturing (longitudinal section). The size of the bacteria was over one micrometer, and the cell wall thickness was about 250 nm. Inner cellwall of lunate spore (arrow 1); Outer cellwall of lunate spore (arrow 2); Adhesion layer, black arrowhead in a, c and g; N, nucleus; M, mitochondria.

**Fig. 4:**
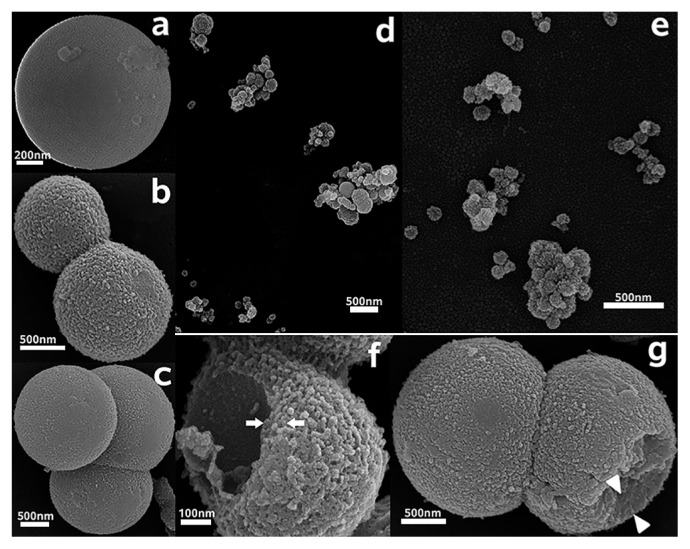
Scanning electron microscope images of endobacteria from *E. vermicola* CBS115803. (a–c) Endobacteria at different division state. (d, e) Endobacteria that are smaller often clumped together. (f, g) Freeze fracture of endobacteria in different size showed a huge difference in thickness of cell wall. (f) The cell wall of a 600 nm diameter endobacterium was about 80 nm thick (arrows). (g) The cell wall of a 2 μm diameter endobacterium was about 250–300 nm thick (arrow heads).

Hyphae and spores both contain coccoid endobacteria in the fungal cytoplasm (TEM, Fig. 3). The purified bacterial symbionts were also coccoid when inspected under SEM (Fig. 4). The morphological characteristics of the endobacteria were clearly revealed. The present state of endobacteria had a single (Fig. 4a), two (Fig. 4b), three (Fig. 4c), or multiple clusters (Fig. 3c, 4d and 4e). Larger endobacteria were about 1–2 μm in diameter, and had a very thick cell wall of approximately 250 nm (Fig. 3f), which may be a dormant state. Microscopic observations revealed that this typically occurred within old hyphae. Reproductive endobacteria that thrived were smaller, typically less than 350 nm, and their cell wall was thinner at approximately 80 nm (Fig. 4f). This was generally the case in germination parts and young hyphae. A Gram-negative cell wall is only a few nanometers thick, while a Gram-positive cell wall is 30–100 nm thick (48). The cell wall of endobacteria inside old hyphae of *E. vermicola* was very different from that of common bacteria.

In the process of SEM observations of endobacteria, some endobacteria were found to have firmly attached stiff fiber structural components that were 25–35 nm in diameter. These fiber components may be microtubules.

### Endobacteria migration before new septal formation

**Fig. 5:**
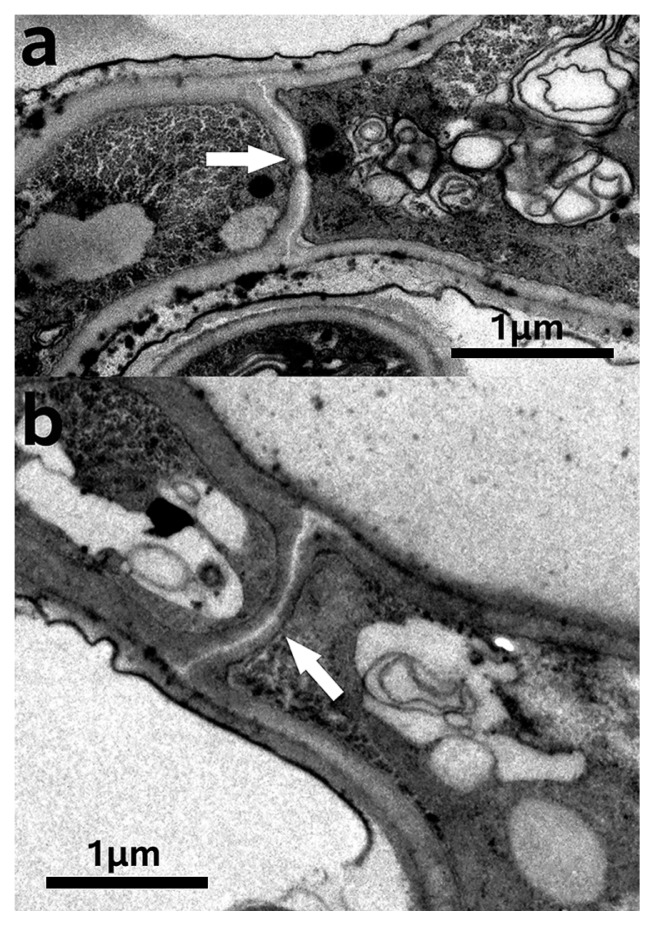
Septa of *E. vermicola* CBS115803. (a) simple pore septum. Septal pore (white arrow); (b) Septal formation initial (white arrow).

Aseptate fungi have been suggested to facilitate the movement of endobacteria along ‘hyphal highways’ (11). The fungi of *E. vermicola* are septate (Fig. S5). It has not yet been clarified whether septa are an obstacle to endobacterial migration inside fungi. We herein demonstrated that the endobacteria of *E. vermicola* migrated toward the fungal growth point (Fig. S4). At the same time, endobacteria always occupied the space of the growing tip (Fig. S5) and emerged at the hyphae tip before septa completed formation (Fig. 3d and 5b), indicating the migration of endobacteria in *E. vermicola* was unblocked by septa.

### Endobacteria and lunate sporulation and germination

Lunate spores are distinctive. The particularity of lunate spores is that they not only harbor endobacteria, they also possess a two-layer intact membrane envelope and thick adhesion layer (approximately 150 nm) on a concave surface that is important for attacking the PWN (Fig. 3a). Lunate conidia germinate markedly faster than cylindrical conidia on water agar (Fig. S6). The formation process of lunate spores is of interest. The commencement of lunate sporulation as well as maturing lunate spores was captured by ultrathin sections and TEM (Fig. 3e and 3f). In Fig. 3e, the appearance of the two-layer envelope during the start of lunate sporulation was clearly observed. Endobacteria existed between the two-layer envelope at this stage. When lunate spores matured, the inner and outer membrane envelopes and adhesion layer had already formed, and endobacteria and fungal nuclei were enclosed in the inner membrane wall.

### Distribution of endobacteria

**Fig. 6:**
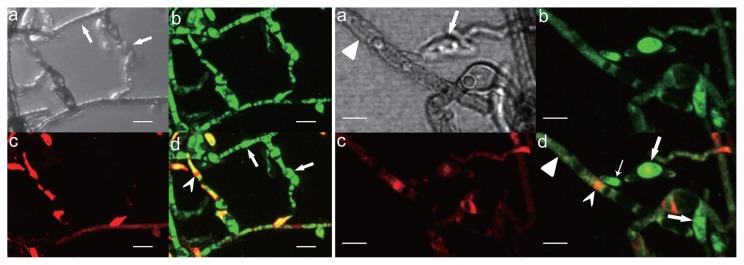
Nucleic acid staining of hyphae of *E. vermicola* CBS115803 using LIVE/DEAD BacLight Bacterial Viability Kits. Left group: stained on young hyphae and conidiogenous cell. Endobacteria were seen as bright green fluorescent signal (d, indicated by arrows). Fungal nuclei were seen as red fluorescent (dovetail arrow head). Right group: stained on old hyphae and spores. Endobacteria in spores are seen as bright green fluorescent (broad arrow, lunate spore; slender arrow, cylindrical conidia), but endobacteria fluorescent signal were weak in old hyphae (arrowhead). (a) Transmitted light. (b) SYTO 9, 480/500 nm. (c) propidium iodide, 490/635 nm. (d) superimposed image. Scale bar, 3 μm.

**Fig. 7:**
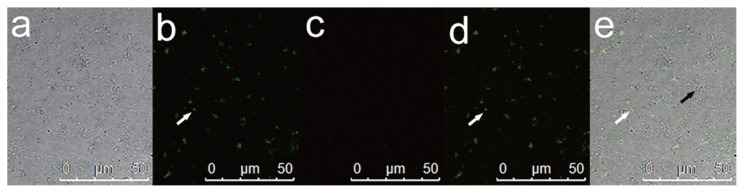
Nucleic acid staining of purified endobacteria from *E. vermicola* CBS115803 using LIVE/DEAD BacLight Bacterial Viability Kits. (a) Transmitted light. (b) SYTO 9 dark field, 480/500 nm. (c) propidium iodide, dark field, 490/635 nm. (d) b and c superimposed image. (e) b, c and d superimposed image. The white arrow indicates fluorescence-stained endobacteria, the black arrow indicates endobacteria without fluorescence.

LIVE/DEAD BacLight Bacterial Viability Kits (Molecular Probes) provide a novel two-color fluorescence assay of bacterial viability. The double labels are SYTO 9 (green-fluorescent) and propidium iodide (red-fluorescent). Using this kit, we stained the axenic hyphae and spores of *E. vermicola* and purified endobacteria. Endobacteria with intact cell membranes stain fluorescent green, whereas fungal nuclei stain fluorescent red because of nuclear pores. A strong green fluorescence signal was noted in the young hyphae and spores of endobacteria. In contrast, the green fluorescence signal was weak in older hyphae (Fig. 6) due to the presence of fewer bacteria. The fluorescence staining of purified endobacteria revealed that some endobacteria have fluorescence signals (Fig. 7 white arrow), whereas others do not (Fig. 7 black arrow).

## Discussion

By using a combination of molecular, morphological, and phylogenetic analyses, this study demonstrated the existence of endobacteria within biocontrol fungal *E. vermicola*, which attacks the PWN, rather than exogenous bacterial contamination. And the intracellular bacteria are enclosed in the so-called endospore-like apparatus discovered by Liou *et al.* presented in lunate conidia of *E. vermicola* through morphological observations (25).

The results of the cultivation and elimination of endobacteria suggested that bacterial growth strongly depended on its host fungus, which appears to substantiate the essential symbiotic relationship. The endobacteria inside *E. vermicola* are vertically transmitted from one generation of *E. vermicola* to the next by sporulation (Fig. S3a). Endobacteria in *E. vermicola* spores grow, multiply, and migrate along with the germination of spores (Fig. S3b).

Fluorescent staining (Fig. 6 and S5) indicated that endobacteria were densely distributed at areas where fungal growth was vigorous. The endobacteria sample was scattered before treatment and observation, and SEM showed that larger endobacteria were present individually, whereas smaller endobacteria frequently clustered together (Fig. 4d and 4e). Clustered endobacteria may be actively dividing and, thus, may not have completely separated, indicating that active cell division often occurred in young thalli. However, further observations of bacterial dynamics in hyphae including cell division are needed. The absence of a fluorescent signal in larger endobacteria was attributed to the thick cell wall. Smaller endobacteria have a thinner cell wall that SYTO9 has the ability to penetrate. Therefore, endobacteria in old and young hyphae differed in cell size, cell wall thickness, the degree of reproduction, and activity, which indicates that the growth of the symbiont and host is synergistic.

TEM, SEM, and FISH results showed that these endobacteria had a coccoid morphology. The closest relative to endobacteria is free-living Gram-negative *P. stutzeri*, which has a bacillary morphology. Although both have highly identical 16S rRNA, their morphologies markedly differ. The genome of obligate endobacteria shrink, and, thus, some genes involved in cell wall biosynthesis are lost or mutated, resulting in the formation of spherical cells from rod-shaped cells (28).

Most endobacteria inside fungi discovered to date have been affiliated with the classes *Gammaproteobacteria*, *Betaproteobacteria*, *Mollicutes*, and *Bacili* (phyla *Firmicutes* and *Proteobacteria*), in addition to the classes *Actinobacteria* and *Alphaproteobacteria* (3, 10, 17, 19, 45, 47). *Pseudomonas* spp. as endobacteria have been identified inside the sporocarps of *Tuber borchii* and *Suillus grevillei*, and some ectomycorrhiza of Scots pine (*Pinus sylvestris*) (15, 52). Additionally, in the present study we discovered it hosted in biocontrol fungal *E. vermicola*. *Pseudomonas* is a genus of very ubiquitous bacteria. *Pseudomonas* species occupy a range of habitats from various soil and water environments to plant and animal tissues, and even fungi cells, and survive as saprophytes, parasites, and mutualisms.

Previous studies reported that it is easier for endobacteria to migrate in aseptate hyphae because the movement of bacteria is not physically restricted (11, 40). The mechanisms by which endobacteria move between two adjacent host cells in septate fungi like *E. vermicola* have not yet been elucidated in detail. We herein propose the following: endobacteria pass through septal pores freely. According to TEM observations, *E. vermicola* belongs to the simple septum category (Fig. 5). These septa possess relatively large central septal pores of 0.05–0.5 μm in diameter, through which cytoplasmic organelles and even nuclei may migrate (16). Depending on their size, endobacteria may migrate freely across the septal pore if only physical factors are considered. A previous study demonstrated that the endobacteria of *Laccaria bicolor* were capable of passing through the dolipore holes (5). These findings suggested that endobacteria migrate from cell to cell through dolipores, similar to organelles (51). Another hypothesis is that endobacteria migrate to the tips of young hypha before septum formation, and this was confirmed by the results shown in Fig. 3d. The transmission of endobacteria toward the direction of growth (Fig. S4) ensures that spores always contain endobacteria. Therefore, the integration of both of these conditions indicates that the septa of fungi are not barriers to endobacterial movement.

Endobacteria replicate and move within their fungal host along with hyphal elongation. However, how endobacteria without flagella migrate inside fungi has not yet been established. The diffusion of large molecules is severely restricted in the cytoplasm (12). A herpes simplex virus (HSV) capsid has been estimated to take 231 years to diffusionally translocate 1 cm in the axonal cytoplasm (12). We demonstrated that microtubules serve as tracks for endobacteria transport (Fig. S7); however, further experiments are warranted. Previous studies revealed that intracellular *Salmonella enterica* and viruses move along microtubules (1, 12). Although further evidence is needed, we propose that endobacteria use host microtubules to move and that the microtubule network provides a transportation aid for the movement of endobacteria within cells.

Endobacteria were directly embedded in the fungal cytoplasm, and occupied a considerable portion of the cytoplasmic volume, which may be significant for metabolic exchange with the host (7). Endobacteria present between two layers of the envelope may produce and secrete enzymes essential for adhesion to or the penetration of the nematode’s body wall, which comprises substances between the two layers of the envelope of a mature lunate spore. The harbored endobacteria make *E. vermicola* very different from other nematophagous fungi (22, 24, 26, 30, 41, 57), and this may result in a distinctive nematophagous mechanism for *E. vermicola*. Therefore, endobacteria may play prominent roles in shaping the formation of lunate spores, which influences nematocidal function, thereby contributing to further ecological success.

Endobacteria within *E. vermicola* may play important roles in the *E. vermicola* infection process. Our results provide a fundamental understanding of the endobacteria inside *E. vermicola*, the biological control fungus, and offer new insights into the tripartite association of endobacteria, fungi, and PWN. These results raise questions on the effects of endobacteria on the biology, ecology, and evolution of biological control fungal hosts, and add to the important and very interesting field of host-symbiont interactions.

## Supplementary Material



## References

[1] Abrahams G.L., Hensel M. (2006). Manipulating cellular transport and immune responses: Dynamic interactions between intracellular *Salmonella enterica* and its host cells. Cell Microbiol.

[2] Archibald J. (2014). One Plus One Equals One: Symbiosis and the Evolution of Complex Life.

[3] Barbieri E., Potenza L., Rossi I., Sisti D., Giomaro G., Rossetti S., Beimfohr C., Stocchi V. (2000). Phylogenetic characterization and *in situ* detection of a *Cytophaga-Flexibacter-Bacteroides* phylogroup bacterium in *Tuber borchii* vittad. Ectomycorrhizal mycelium. Appl Environ Microbiol.

[4] Bertaux J., Schmid M., Prevost-Boure N.C., Churin J.L., Hartmann A., Garbaye J., Frey-Klett P. (2003). *In Situ* identification of intracellular bacteria related to *Paenibacillus* spp. in the mycelium of the ectomycorrhizal fungus *Laccaria bicolor* S238N. Appl Environ Microbiol.

[5] Bertaux J., Schmid M., Hutzler P., Hartmann A., Garbaye J., Frey-Klett P. (2005). Occurrence and distribution of endobacteria in the plant-associated mycelium of the ectomycorrhizal fungus *Laccaria bicolor* S238N. Environ Microbiol.

[6] Bianciotto V., Bandi C., Minerdi D., Sironi M., Tichy H.V., Bonfante P. (1996). An obligately endosymbiotic mycorrhizal fungus itself harbors obligately intracellular bacteria. Appl Env Microbiol.

[7] Bianciotto V., Lumini E., Bonfante P., Vandamme P. (2003). “*Candidatus* Glomeribacter gigasporarum” gen. nov., sp. nov., an endosymbiont of arbuscular mycorrhizal fungi. Int J Syst Evol Microbiol.

[8] Bonfante P., Balestrini R., Mendgen K. (1994). Storage and secretion processes in the spore of *Gigaspora Margarita* becker and hall as revealed by high-pressure freezing and freeze-substitution. New Phytol.

[9] Chu W.H., Dou Q., Chu H.L., Wang H.H., Sung C.K., Wang C.Y. (2015). Research advance on *Esteya vermicola*, a high potential biocontrol agent of pine wilt disease. Mycol Prog.

[10] Desirò A., Salvioli A., Ngonkeu E.L., Mondo S.J., Epis S., Faccio A., Kaech A., Pawlowska T.E., Bonfante P. (2014). Detection of a novel intracellular microbiome hosted in arbuscular mycorrhizal fungi. ISME J.

[11] Desirò A., Faccio A., Kaech A., Bidartondo M.I., Bonfante P. (2015). *Endogone*, one of the oldest plant-associated fungi, host unique Mollicutes-related endobacteria. New Phytol.

[12] Döhner K., Nagel C.H., Sodeik B. (2005). Viral stop-and-go along microtubules: Taking a ride with dynein and kinesins. Trends Microbiol.

[13] Ferrari J., Vavre F. (2011). Bacterial symbionts in insects or the story of communities affecting communities. Philos Trans R Soc Lond B Biol Sci.

[14] Futai K. (2013). Pine wood nematode, *Bursaphelenchus xylophilus*. Annu Rev Phytopathol.

[15] Gazzanelli G., Malatesta M., Pianetti A., Baffone W., Stocchi V., Citterio B. (1999). Bacteria associated to fruit bodies of the ectomycorrhizal fungus *Tuber borchii* Vittad. Symbiosis.

[16] Held M., Binz M., Edwards C., Nicolau D.V. (2009). Dynamic behaviour of fungi in microfluidics: a comparative study. Proc SPIE.

[17] Hoffman M.T., Arnold A.E. (2010). Diverse bacteria inhabit living hyphae of phylogenetically diverse fungal endophytes. Appl Environ Microbiol.

[18] Hoffmeister M., Martin W. (2003). Interspecific evolution: Microbial symbiosis, endosymbiosis and gene transfer. Environ Microbiol.

[19] Izumi H., Anderson I.C., Alexander I.J., Killham K., Moore E.R.B. (2006). Endobacteria in some ectomycorrhiza of Scots pine (*Pinus sylvestris*). FEMS Microbiol Ecol.

[20] Kai K., Furuyabu K., Tani A., Hayashi H. (2012). Production of the quorum-sensing molecules N-acylhomoserine lactones by endobacteria associated with *Mortierella alpina* A-178. ChemBioChem.

[21] Lackner G., Partida-Martinez L.P., Hertweck C. (2009). Endofungal bacteria as producers of mycotoxins. Trends Microbiol.

[22] Lai Y., Liu K., Zhang X. (2014). Comparative genomics and transcriptomics analyses reveal divergent lifestyle features of nematode endoparasitic fungus *Hirsutella minnesotensis*. Genome Biol Evol.

[23] Lalucat J., Bennasar A., Bosch R., García-Valdés E., Palleroni N.J. (2006). Biology of *Pseudomonas stutzeri*. Microbiol Mol Biol Rev.

[24] Larriba E., Jaime M.D.L.A., Carbonell-Caballero J., Conesa A., Dopazo J., Nislow C., Martín-Nieto J., Lopez-Llorca L.V. (2014). Sequencing and functional analysis of the genome of a nematode egg-parasitic fungus, *Pochonia chlamydosporia*. Fungal Genet Biol.

[25] Liou J.Y., Shih J.Y., Tzean S.S. (1999). *Esteya*, a new nematophagous genus from Taiwan, attacking the pinewood nematode (*Bursaphelenchus xylophilus*). Mycol Res.

[26] Liu K., Zhang W., Lai Y., Xiang M., Wang X., Zhang X., Liu X. (2014). *Drechslerella stenobrocha* genome illustrates the mechanism of constricting rings and the origin of nematode predation in fungi. BMC Genomics.

[27] McCutcheon J.P. (2013). Genome evolution: A bacterium with a napoleon complex. Curr Biol.

[28] McCutcheon J.P., Moran N. (2011). Extreme genome reduction in symbiotic bacteria. Nat Rev Microbiol.

[29] McFall-Ngai M., Hadfield M.G., Bosch T.C.G. (2013). Animals in a bacterial world, a new imperative for the life sciences. Proc Natl Acad Sci USA.

[30] Meerupati T., Andersson K.M., Friman E., Kumar D., Tunlid A., Ahrén D. (2013). Genomic mechanisms accounting for the adaptation to parasitism in nematode-trapping fungi. PLoS Genet.

[31] Miller M.A., Pfeiffer W., Schwartz T. (2010). Creating the CIPRES Science Gateway for inference of large phylogenetic trees. Gatew Comput Environ Work (GCE).

[32] Mondo S.J., Toomer K.H., Morton J.B., Lekberg Y., Pawlowska T.E. (2012). Evolutionary stability in a 400-million year old heritable facultative mutualism. Evolution (N Y).

[33] Moran N.A., Yun Y. (2015). Experimental replacement of an obligate insect symbiont. Proc Natl Acad Sci USA.

[34] Mosse B. (1970). Honey-coloured, sessile Endogone spores. Arch Microbiol.

[35] Naito M., Morton J.B., Pawlowska T.E. (2015). Minimal genomes of mycoplasma-related endobacteria are plastic and contain host-derived genes for sustained life within Glomeromycota. Proc Natl Acad Sci USA.

[36] Naumann M., Schüssler A., Bonfante P. (2010). The obligate endobacteria of arbuscular mycorrhizal fungi are ancient heritable components related to the *Mollicutes*. ISME J.

[37] Oliver K.M., Smith A.H., Russell J.A. (2014). Defensive symbiosis in the real world—advancing ecological studies of heritable, protective bacteria in aphids and beyond. Funct Ecol.

[38] Partida-Martinez L.P., Hertweck C. (2005). Pathogenic fungus harbours endosymbiotic bacteria for toxin production. Nature.

[39] Partida-Martinez L.P., De Looß C.F., Ishida K., Ishida M., Roth M., Buder K., Hertweck C. (2007). Rhizonin, the first mycotoxin isolated from the zygomycota, is not a fungal metabolite but is produced by bacterial endosymbionts. Appl Environ Microbiol.

[40] Partida-Martinez L.P., Monajembashi S., Greulich K.-O.O., Hertweck C. (2007). Endosymbiont-dependent host reproduction maintains bacterial-fungal mutualism. Curr Biol.

[41] Prasad P., Varshney D., Adholeya A. (2015). Whole genome annotation and comparative genomic analyses of bio-control fungus *Purpureocillium lilacinum*. BMC Genomics.

[42] Pruesse E., Quast C., Knittel K., Fuchs B., Ludwig W. (2007). SILVA: a comprehensive online resource for quality checked and aligned ribosomal RNA sequence data. Nucleic Acids Res.

[43] Ronquist F., Teslenko M., van der Mark P., Ayres D.L., Darling A., Hohna S., Larget B., Liu L., Suchard M.A., Huelsenbeck J.P. (2012). Efficient Bayesian phylogenetic inference and model choice across a large model space. Syst Biol.

[44] Salvioli A., Ghignone S., Novero M., Navazio L., Venice F., Bagnaresi P., Bonfante P. (2015). Symbiosis with an endobacterium increases the fitness of a mycorrhizal fungus, raising its bioenergetic potential. ISME J.

[45] Sato Y., Narisawa K., Tsuruta K., Umezu M., Nishizawa T., Tanaka K., Yamaguchi K., Komatsuzaki M., Ohta H. (2010). Detection of betaproteobacteria inside the mycelium of the fungus *Mortierella elongata*. Microbes Environ.

[46] Schloss P.D., Westcott S.L., Ryabin T. (2009). Introducing mothur: open-source, platform-independent, community-supported software for describing and comparing microbial communities. Appl Environ Microbiol.

[47] Sharma M., Schmid M., Rothballer M. (2008). Detection and identification of bacteria intimately associated with fungi of the order *Sebacinales*. Cell Microbiol.

[48] Silhavy T.J., Kahne D., Walker S. (2010). The bacterial cell envelope. Cold Spring Harb Perspect Biol.

[49] Stamatakis A. (2014). RAxML version 8: A tool for phylogenetic analysis and post-analysis of large phylogenies. Bioinformatics.

[50] Torres-Cortés G., Ghignone S., Bonfante P., Schüßler A. (2015). Mosaic genome of endobacteria in arbuscular mycorrhizal fungi: Transkingdom gene transfer in an ancient mycoplasma-fungus association. Proc Natl Acad Sci USA.

[51] van Peer A.F., Wang F., van Driel K.G.A., de Jong J.F., van Donselaar E.G., Müller W.H., Boekhout T., Lugones L.G., Wösten H.A.B. (2010). The septal pore cap is an organelle that functions in vegetative growth and mushroom formation of the wood-rot fungus *Schizophyllum commune*. Environ Microbiol.

[52] Varese G.C., Portinaro S., Trotta A., Scannerini S., Luppi-Mosca A., Martinotti M.G. (1996). Bacteria associated with *Suillus grevillei* sporocarps and ectomycorrhizae and their effects on *in vitro* growth of the mycobiont. Symbiosis.

[53] Wang C.Y., Fang Z.M., Sun B.S., Gu L.J., Zhang K.Q., Sung C.K. (2008). High infectivity of an endoparasitic fungus strain, *Esteya vermicola*, against nematodes. J Microbiol.

[54] Wang C.Y., Wang Z., Fang Z.M., Zhang D.L., Gu L.J., Liu L., Sung C.K. (2010). Attraction of pinewood nematode to endoparasitic nematophagous fungus *Esteya vermicola*. Curr Microbiol.

[55] Wang C.Y., Fang Z.M., Wang Z., Zhang D.L., Gu L.J., Lee M.R., Liu L., Sung C.K. (2011). Biological control of the pinewood nematode *Bursaphelenchus xylophilus* by application of the endoparasitic fungus *Esteya vermicola*. BioControl.

[56] Wernegreen J.J. (2012). Endosymbiosis. Curr Biol.

[57] Yang J., Wang L., Ji X. (2011). Genomic and proteomic analyses of the fungus *arthrobotrys oligospora* provide insights into nematode-trap formation. PLoS Pathog.

