# Supplementary material for: Esteya Vermicola, a Nematophagous Fungus Attacking the Pine Wood Nematode, Harbors a Bacterial Endosymbiont Affiliated with Gammaproteobacteria
Source: Microbes Environ. 2017 Sep 27;32(3):201–9. doi: 10.1264/jsme2.ME16167 (PMC5606689; doi:10.1264/jsme2.ME16167)

Supporting information

Figure S1

PCR product from fungal gDNA (CBS 115803) using generic bacterial 16S primers 27f/1492r. M, marker; Lane 1 and lane 2 is PCR product of CBS 115803 gDNA. Lane 3 is positive control, PCR product of gDNA from *Escherichia Coli*. Lane 4 is the negative control using nuclease free water.

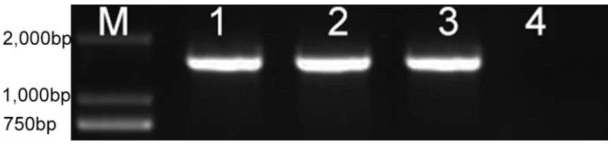

Figure S2

PCR product from fungal gDNA (ATCC 74485, CBS100821, CNU120806 and CBS 115803) using generic bacterial 16S primers 27f/1492r. M, marker; Lane 1, ATCC 74485; lane 2, CBS100821; Lane 3, CNU120806; lane 4, CBS 115803; Lane 5 is positive control, PCR product of gDNA from *Escherichia Coli*. Lane 6 is the negative control using nuclease free water. 7, 8, 9 and 10 is PCR product from the filtrate of mycelium and spores (ATCC 74485, CBS100821, CNU120806 and CBS 115803, respectively).

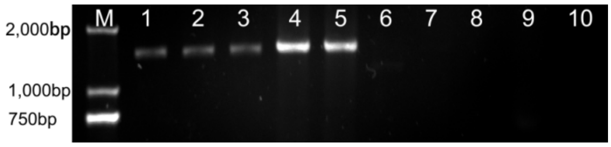

Figure S3

a, vertical transmission of endobacteria inside *E. vermicola*. A lunate spore (above) germinated and produced a new lunate conidia (below), and endobacteria was transmitted vertically synchronously (indicated by arrow). b, growth and proliferation of endobacteria in different germination state of lunate spores. There were many endobacteria in geminating lunate spore. Photographed under Optical microscope. Scale bar = 5μm.

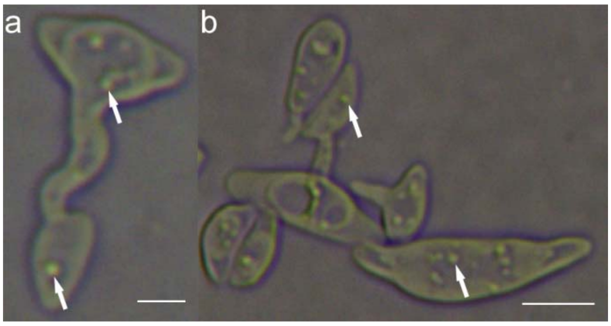

Figure S4

Endobacteria was transmitted toward the direction of growth (indicated by arrow). Photographed under Optical microscope. a, b and c, cylindrical conidia; d, e and f, lunate conidia. Scale bar = 5μm.

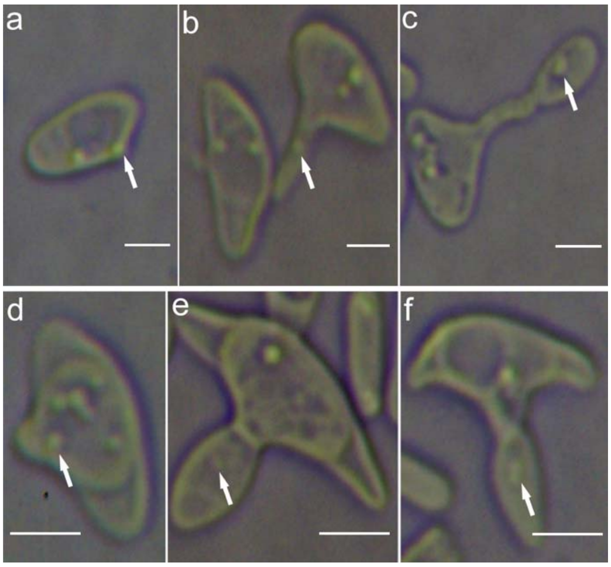

Figure S5

Endobacteria (arrow) in young and old hyphae of EV. There were more than ten endobacteria (250nm) in the hyphae tip cell (a), while in the old hyphae (b) only three bacteria (1-2μm) exist. Photographed under Optical microscope. a, young hyphae, Scale bar = 0.5μm; b, old hyphae, Scale bar = 5μm. Septa of EV (indicated by arrow head).

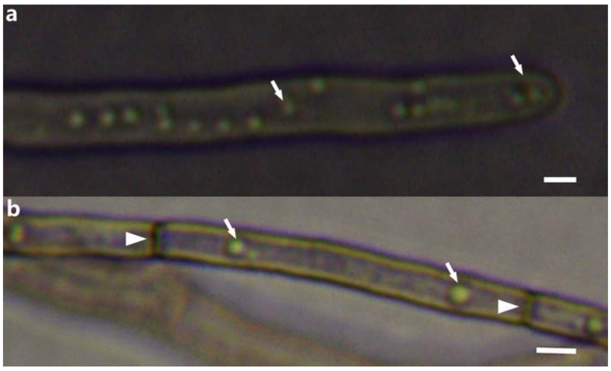

Figure S6

Germination of lunate conidia (white arrow) and cylindrical conidia (black arrow) on water agar (24h). Nearly all lunate spores germinated at 24h. In contrast, cylindrical conidia did not. Scale bar = 50μm.

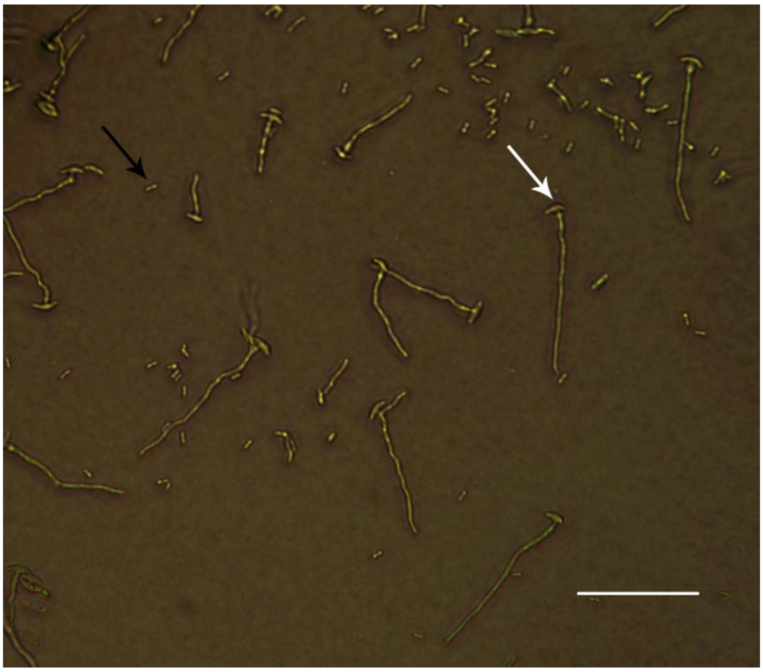

Figure S7

Endobacteria (arrow) attached stiff fiber structural components. They were stiff and can resist bending forces. Here they were identified as microtubules (arrow head).

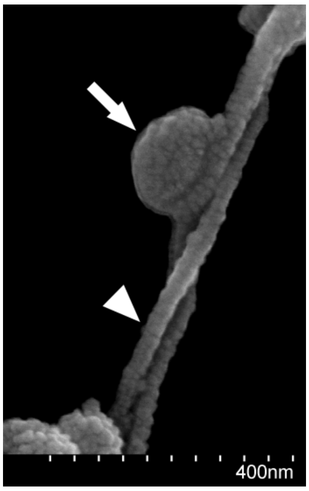

Supplement: Supplementary file 1 [file 32_201_s1.pdf]
